# Risk Factors, Trends, and Financial Impact for 30-Day Unplanned Readmissions in Patients Admitted With Myocarditis and COVID-19: Insights From the Healthcare Cost and Utilization Project (HCUP) Nationwide Readmission Database

**DOI:** 10.7759/cureus.80371

**Published:** 2025-03-10

**Authors:** Dheeraj Kommineni, Priji Prasad Jalaja, Ramakrishna Tumati, Dilip Kumar, Anirban Majumder, Chrishanti Anna Joseph

**Affiliations:** 1 Department of Systems Analytics, Hanker Systems, Chantilly, USA; 2 Department of Surgery, Emory University, Atlanta, USA; 3 Department of Software Application &amp; Engineering, Intel, Beaverton, USA; 4 Department of Engineering, Snowflake Inc, San Mateo, USA; 5 Research, Amazon Science Education, Scottsdale, USA; 6 Department of Anesthesiology and Perioperative Medicine, University of Pittsburgh, Pittsburgh, USA

**Keywords:** covid-2019, covid and myocarditis, healthcare cost and utilization project, myocarditis, readmission risk

## Abstract

Background:The coronavirus disease 2019 (COVID-19) outbreak was first documented in Wuhan, China, in December 2019. Myocarditis, an inflammatory condition characterized by swelling and thickening of the heart muscle, has been linked to severe COVID-19 cases, contributing to worse clinical outcomes. The SARS-CoV-2 virus enters human cells through angiotensin-converting enzyme 2 (ACE2), and myocardial involvement can result from direct viral invasion, hyperinflammation, and immune-mediated damage. The exact prevalence of myocarditis among COVID-19 patients remains uncertain due to initial diagnostic limitations.

Objective: This study aims to evaluate the risk factors, trends, financial impact, and preventive strategies related to 30-day unplanned hospital readmission in patients diagnosed with both myocarditis and COVID-19.

Methodology: A retrospective analysis was conducted using a nationwide hospital database from 2020. Patients diagnosed with both myocarditis and COVID-19 were identified based on standardized diagnostic coding criteria. Confounding factors were addressed using multivariable logistic regression to adjust for demographics, comorbidities, and hospital characteristics.

Results:After applying inclusion and exclusion criteria, 28,726 patients were included, with 4,896 (17.04%) experiencing hospital readmission within 30 days. Compared to national readmission rates for other cardiovascular conditions, this rate is notably high. The median patient age was 67 years (interquartile range {IQR}: 56-78, p < 0.001). Women accounted for 38.1% of readmitted patients. Medicare was the primary insurer for 60.9% of the total cohort and 61.9% of those readmitted (p < 0.001). The median cost of the initial hospitalization was estimated at USD 56,480.37 (IQR: USD 56,433.13-56,930.00), highlighting the financial burden of these readmissions. Among readmitted patients, the median length of stay was seven days (IQR: 6-7 days). Multivariable logistic regression identified heart failure (adjusted odds ratio {AOR} 2.14, 95% confidence interval {CI}: 1.91-2.41, p < 0.001), chronic kidney disease (AOR 1.87, 95% CI: 1.63-2.14, p < 0.001), and diabetes mellitus (AOR 1.56, 95% CI: 1.38-1.76, p < 0.001) as the most significant comorbidities associated with readmission.

Conclusion: Our study found that the readmission rate of patients with COVID-19 and myocarditis was highest between days 7 and 14, with 42.3% of readmissions occurring in this period. This emphasizes the need for close post-discharge monitoring and timely follow-up appointments to reduce adverse outcomes. Additionally, patients with comorbidities such as heart failure, chronic kidney disease, and diabetes mellitus had a significantly higher risk of readmission, necessitating targeted management strategies. The substantial financial burden of readmissions underscores the need for healthcare system interventions to optimize post-discharge care.

## Introduction

Myocarditis, an inflammatory condition of the myocardium, is a clinically significant disorder that can result in severe cardiac complications, including arrhythmias, cardiogenic shock, and heart failure [1]. It is often triggered by viral infections, autoimmune disorders, or drug reactions, with viral myocarditis being one of the most common causes [2]. The emergence of COVID-19 has introduced new complexities in the epidemiology and management of myocarditis, as growing evidence suggests that SARS-CoV-2 infection can lead to direct myocardial injury, inflammation, and subsequent dysfunction [3]. While some studies have provided clinical evidence of COVID-19-associated myocarditis in hospitalized patients, large-scale clinical trials validating its exact prevalence and mechanisms remain limited. Studies have demonstrated that myocarditis in COVID-19 patients can occur due to several mechanisms, including direct viral invasion of cardiac myocytes, hyperinflammatory responses, and microvascular thrombosis, all of which contribute to increased morbidity and mortality [4]. These mechanisms may result in persistent myocardial dysfunction and residual inflammation, increasing the likelihood of hospital readmission. Given these pathophysiological factors, patients hospitalized with myocarditis, particularly in the setting of COVID-19, may experience a higher risk of complications and hospital readmissions [5].

Hospital readmission within 30 days of discharge is widely recognized as an important healthcare quality metric, reflecting the effectiveness of inpatient care, post-discharge follow-up, and overall disease management [6]. Unplanned readmissions not only pose a significant burden on patients by increasing the likelihood of further complications but also place a strain on healthcare resources, leading to higher costs for both hospitals and insurance providers [7]. In cardiovascular diseases, several factors such as older age, multiple comorbidities, severity of the initial illness, and inadequate transitional care contribute to early readmissions [8]. However, transitional care for myocarditis patients remains suboptimal due to the lack of standardized post-discharge protocols and outpatient follow-up limitations. While extensive research has been conducted on readmission trends in heart failure, acute coronary syndromes, and arrhythmias, myocarditis readmissions have not been widely studied as a benchmark for care quality, making this study a novel contribution to the literature [9]. Understanding the specific risk factors driving 30-day unplanned readmissions in this population is crucial for developing targeted interventions that can improve patient outcomes and reduce healthcare expenditures [10].

The Healthcare Cost and Utilization Project (HCUP) Nationwide Readmission Database (NRD) provides a comprehensive dataset to analyze hospital readmission patterns at a national level [11,12]. By leveraging this database, we can assess the demographic and clinical characteristics associated with increased readmission risk in patients hospitalized with myocarditis, specifically in the presence of COVID-19, without direct comparison to non-COVID-19 cases, as the title and abstract indicate. Additionally, evaluating the financial implications of these readmissions will include direct hospital costs rather than broader economic impacts such as insurance reimbursements.

This study aims to examine the risk factors, trends, and financial impact of 30-day unplanned readmissions among patients admitted with myocarditis and COVID-19, using data from the HCUP NRD. By identifying specific high-risk subgroups based on demographic factors, clinical severity, and comorbidity profiles, the findings will contribute to the development of evidence-based interventions that can enhance patient care, reduce hospital utilization, and alleviate economic burdens on healthcare systems. Specifically, the study aims to inform strategies such as early follow-up care, improved post-discharge monitoring, and enhanced outpatient management to mitigate readmission risks. A deeper understanding of these dynamics will be essential in refining hospital discharge protocols, strengthening outpatient follow-up strategies, and ultimately improving long-term outcomes for myocarditis patients.

## Materials and methods

Study design

A retrospective analysis was conducted using de-identified patient data from the 2020 Nationwide Readmission Database (NRD). Index admissions were identified based on COVID-19 and myocarditis diagnosis codes. As the study utilized de-identified data, it was exempt from Institutional Review Board (IRB) approval. While the NRD is publicly available, access requires an application and adherence to data use agreements.

Data source

The NRD provided data from January 1, 2020, to November 30, 2020, as the December dataset was incomplete at the time of analysis. The NRD is the largest all-patient, all-payer inpatient database in the United States, created and maintained by the Agency for Healthcare Research and Quality for the HCUP. It includes hospitalization data from nonfederal hospitals across 31 geographically diverse states, representing 60.8% of all U.S. hospitalizations and 62.2% of the population. These coverage rates are consistent with previous years, though state participation may introduce selection biases. The NRD allows analysis at both the hospital and patient levels, with up to 40 discharge diagnoses and 25 procedures recorded using the International Classification of Diseases, 10th Revision, Clinical Modification (ICD-10-CM) [13]. Both primary and secondary diagnoses were used to identify COVID-19 and myocarditis cases. Hospitals are categorized by geographic region, teaching status, bed count, ownership, and urban/rural location. The NRD enables weighted analysis to estimate nationwide hospitalizations for a given year.

Patients older than or equal to 18 years old with a primary diagnosis of COVID and myocarditis based on ICD-10-CM codes were eligible to participate. Among the exclusion criteria were the following: (1) under 18 years old, and (2) death data missing from the index admission.

Covariates

Patient demographics for the index admission included gender, age, insurance coverage, median household income, functional status impairment (e.g., mobility limitations or organ dysfunction), and discharge disposition. ICD-10-CM codes were used to identify comorbidities, which were analyzed individually rather than using a specific comorbidity index such as Elixhauser or Charlson. Hospital characteristics, including bed size, ownership (private vs. government), designation (large, small, micropolitan, non-urban), and teaching status, were also collected. ICD-10 procedure codes identified interventions performed during hospitalization. The All Patients Refined Diagnosis Related Group (APR-DRG) was used to assess disease severity. Outcomes were defined as 30-day readmission (any unplanned hospitalization within 30 days of discharge) and no readmission (no hospitalization within this period).

Statistical analysis

IBM SPSS Statistics for Windows, version 23 (IBM Corp., Armonk, NY), was used for all statistical analyses. Before selecting a non-parametric test, normality assumptions were assessed using the Shapiro-Wilk test. The Pearson chi-square test was applied to categorical variables, while the Mann-Whitney U test was used for continuous variables, with no mortality as the reference group, to compare baseline characteristics such as gender, age, weekend versus weekday admissions, household income, payer status, functional status impairment, and likelihood of dying. Multivariable logistic regression was conducted to examine clinical factors associated with 30-day readmission, adjusting for potential confounders such as disease severity (APR-DRG) and pre-existing conditions (individual comorbidities). Additionally, multiple logistic regression models were used to identify independent predictors of mortality, analyzing deaths occurring both during the initial hospitalization and within the readmission period. Results were reported as odds ratios (OR) with 95% confidence intervals (CI).

Patient and public involvement

This study utilized the NRD, a publicly accessible all-payer inpatient healthcare readmission database in the United States, derived from the AHRQ HCUP. As the NRD contains de-identified data, no direct patient involvement, patient-reported outcomes, or perspectives were included in this study.

Data availability statement

The Nationwide Readmissions Database (NRD) is a publicly accessible, all-payer inpatient care database in the United States, containing data from over 17 million hospital visits. It provides comprehensive insights into hospitalization patterns, facilitating large-scale healthcare research. The data is freely available and openly accessible without licensing fees or institutional restrictions.

## Results

Following the application of inclusion and exclusion criteria, a total of 28,666 patients were selected for analysis. Among them, 4,391 individuals (15.3%) experienced readmission within 30 days. Table 1 outlines the baseline demographic and clinical characteristics of the study population. The median age of the patients was 67 years (interquartile range {IQR}: 56-78, p < 0.001). Women comprised 38.1% of those who were readmitted. Medicare served as the primary insurance provider for 60.9% of the total cohort and 61.9% of those readmitted (p < 0.001). The median cost of the initial hospitalization was USD 56,480.37 (IQR: USD 56,433.13-56,930.00).

**Table 1:** Baseline characteristics of patients who survived index admission LOS = length of stay; APRDRG-SOI = all patients refined diagnosis-related group – severity of illness

Characteristics	Total population in study	No readmission (24,275)	30-day readmission	Test statistic	Effect size	P value
	(n=28,666)		(n=4,391 (13.1%)			
Age (in years), median (IQR)	67.00 (56-78)	67.00 (56-78)	67.00 (56-78)	U = 1.2×10⁹	-	< .001
Women	9435 (38.9%)	1487 (33.9%)	10922 (38.1%)	χ² = 25.6 (df=1)	Phi = 0.03	< .001
Elective	482 (2.0%)	63 (1.4%)	545 (1.9%)	χ² = 5.9 (df=1)	Phi = 0.02	0.015
Weekend admission	6036 (24.9%)	1150 (26.2%)	7186 (25.1%)	χ² = 3.5 (df=1)	Phi = 0.01	0.063
Insurance status	-	-	-	χ² = 120.4 (df=5)	Cramer’s V = 0.05	< .001
Medicare	14747 (60.9%)	2959 (67.5%)	17706 (61.9%)	-	-	-
Medicaid	3137 (13.0%)	635 (14.5%)	3772 (13.2%)	-	-	-
Private	4947 (20.4%)	590 (13.5%)	5537 (19.4%)	-	-	-
Self-pay	608 (2.5%)	84 (1.9%)	692 (2.4%)	-	-	-
No charge	70 (0.3%)	<10 (0.1%)	76 (0.3%)	-	-	-
Other	711 (2.9%)	110 (2.5%)	821 (2.9%)		-	-
Cost of hospitalization in US$, median (IQR)	56480.37 (56433.13-56930.00)	-	-	U = 8.9×10⁸	-	< .001
LOS, median (IQR)	7 days (6-7)	6 days (6-7)	7 days (6-7)	U = 1.0×10⁹	-	-
Quartile of median household income	-	-	-	χ² = 15.2 (df=3)	Cramer’s V = 0.02	< .001
0-25th	8823 (36.7%)	1687 (38.8%)	10510 (37.0%)	-	-	-
26th-50th	6857 (28.5%)	1253 (28.8%)	8110 (28.6%)	-	-	-
51st-75th	4847 (20.2%)	859 (19.8%)	5706 (20.1%)	-	-	-
76th-100th	3511 (14.6%)	549 (12.6%)	4060 (14.3%)	-	-	-
APRDRG-SOI, likelihood of dying	-	-	-	χ² = 11.3 (df=3)	Cramer’s V = 0.02	0.01
Minor	17 (0.1%)	<10 (0.1%)	20 (0.1%)	-	-	-
Moderate	616 (2.5%)	66 (1.5%)	682 (2.4%)	-	-	-
Major	12465 (51.3%)	2263 (51.5%)	14728 (51.4%)	-	-	-
Extreme	11177 (46.0%)	2060 (46.9%)	13237 (46.2%)	-	-	-
APRDRG-SOI, loss of function	-	-	-	χ² = 2.7 (df=3)	Cramer’s V = 0.01	0.419
Minor (includes cases with no comorbidity or complications)	10 (0.0%)	<10 (0.1%)	16 (0.1%)	-	-	-
Moderate	264 (1.1%)	29 (0.7%)	293 (1.0%)	-	-	-
Major	13559 (55.9%)	2414 (55.0%)	15973 (55.7%)	-	-	-
Extreme	10442 (43.0%)	1942 (44.2%)	12384 (43.2%)	-	-	-
Hospital bed size	-	-	-	χ² = 3.8 (df=2)	Cramer’s V = 0.01	0.151
Small	4555 (18.8%)	823 (18.7%)	5378 (18.8%)	-	-	-
Medium	6137 (25.3%)	1162 (26.5%)	7299 (25.5%)	-	-	-
Large	13583 (56.0%)	2407 (54.8%)	15990 (55.8%)	-	-	-
Control/ownership of hospital	-	-	-	χ² = 45.1 (df=2)	Cramer’s V = 0.04	< .001
Government	2653 (9.3%)	483 (1.7%)	3136 (10.9%)	-	-	-
Private non-profit	18601 (76.6%)	3247 (73.9%)	21848 (76.2%)	-	-	-
Private for-profit	3021 (12.4%)	661 (15.1%)	3682 (12.8%)	-	-	-
Hospital designation	-	-	-	χ² = 12.9 (df=3)	Cramer’s V = 0.02	0.004
Large metropolitan, ≥1 million residents	14076 (58.0%)	2627 (59.8%)	16703 (58.3%)	-	-	-
Small metropolitan, ≤1 million residents	8194 (33.8%)	1426 (32.5%)	9620 (33.6%)	-	-	-
Micropolitan	1617 (6.7%)	288 (6.6%)	1905 (6.6%)	-	-	-
Non-urban residual	390 (1.6%)	50 (1.1%)	440(1.5%)	-	-	-
Hospital teaching status	-	-	-	-	-	-
Metropolitan, non-teaching	4112 (16.9%)	878 (20.0%)	4990 (17.4%)	-	-	-
Metropolitan, teaching	18157 (74.8%)	3175 (72.3%)	21332 (74.4%)	-	-	-
Non-metropolitan	2006 (8.3%)	338 (7.7%)	2344 (8.2%)	-	-	-
Discharge location	-	-	-	χ² = 120.7 (df=4)	Cramer’s V = 0.05	< .001
Home self-care	11749 (48.4%)	1972 (44.9%)	13721 (47.9%)	-	-	-
Home healthcare	347 (1.4%)	63 (1.4%)	410 ((1.4%)	-	-	-
Transfer to short-term hospital	6764 (27.9%)	1213 (27.6%)	7977 (27.8%)	-	-	-
Transfer to other care facility	5043 (20.8%)	1000 (22.8%)	6043 (21.1%)	-	-	-
Against medical advice	335 (1.4%)	144 (3.3%)	479 (1.7%)	-	-	-

Table 2 summarizes the comorbid conditions observed at both initial admission and during 30-day readmission. Patients who were readmitted had a median hospital stay of seven days (IQR: 6-7 days).

**Table 2:** Comorbidities of patients who survived index admission AMI = acute myocardial infarction; HTN = hypertension; AKI = acute kidney injury; CKD = chronic kidney disease; CVA = cerebrovascular accident; TIA = transient ischemic attack; PAD = peripheral arterial disease; GI = gastrointestinal † Fisher’s exact test was used due to small expected cell counts (<5 cases) to ensure statistical accuracy

Comorbidities	Total (n=28,666)	No readmission (n=24,275)	30-day readmission (n=4,391)	Test statistic	Effect size	P value
Acute MI	3,591 (12.5%)	3,027 (12.5%)	564 (12.8%)	χ² = 0.5 (df=1)	Phi = 0.004	0.489
Tobacco abuse	7 (0.0%)	7 (0.0%)	0 (0.0%)	Fisher’s exact test†	—	0.605†
Alcohol abuse	911 (3.2%)	733 (3.0%)	178 (4.1%)	χ² = 15.2 (df=1)	Phi = 0.02	<0.001
Lipid disorders	14,933 (52.1%)	12,621 (52.0%)	2,312 (52.6%)	χ² = 0.6 (df=1)	Phi = 0.005	0.431
Hypertension (HTN)	4,496 (15.7%)	3,996 (16.5%)	500 (11.4%)	χ² = 45.1 (df=1)	Phi = 0.04	<0.001
Diabetes	13,395 (46.7%)	11,140 (45.9%)	2,255 (51.3%)	χ² = 32.7 (df=1)	Phi = 0.03	<0.001
Obesity	7,849 (27.4%)	6,709 (27.6%)	1,140 (26.0%)	χ² = 5.2 (df=1)	Phi = 0.02	0.023
Heart failure	20,288 (70.8%)	16,899 (69.6%)	3,389 (77.2%)	χ² = 85.7 (df=1)	Phi = 0.06	<0.001
Chronic lung disease	18,757 (65.4%)	15,859 (65.3%)	2,898 (66.0%)	χ² = 0.7 (df=1)	Phi = 0.005	0.408
Acute kidney injury (AKI)	11,312 (39.5%)	9,571 (39.4%)	1,741 (39.6%)	χ² = 0.07 (df=1)	Phi = 0.002	0.789
Chronic kidney disease (CKD)	11,448 (39.9%)	9,242 (38.1%)	2,206 (50.2%)	χ² = 210.3 (df=1)	Phi = 0.09	<0.001
Non-cardiac chest pain	504 (1.8%)	397 (1.6%)	107 (2.4%)	χ² = 14.2 (df=1)	Phi = 0.02	<0.001
Valvular heart disease	2,147 (7.5%)	1,743 (7.2%)	404 (9.2%)	χ² = 22.4 (df=1)	Phi = 0.03	<0.001
Cerebrovascular accident (CVA)	602 (2.1%)	526 (2.2%)	76 (1.7%)	χ² = 3.3 (df=1)	Phi = 0.01	0.069
Transient ischemic attack (TIA)	85 (0.3%)	73 (0.3%)	12 (0.3%)	χ² = 0.02 (df=1)	Phi = 0.003	0.88
Peripheral arterial disease (PAD)	1,229 (4.3%)	1,019 (4.2%)	210 (4.8%)	χ² = 3.1 (df=1)	Phi = 0.01	0.08
Pulmonary circulatory disorders	2,887 (10.1%)	2,348 (9.7%)	539 (12.3%)	χ² = 25.4 (df=1)	Phi = 0.03	<0.001
GI bleed	994 (3.5%)	811 (3.3%)	183 (4.2%)	χ² = 7.4 (df=1)	Phi = 0.02	0.007
Thyroid disorders	4,204 (14.7%)	3,557 (14.7%)	647 (14.7%)	χ² = 0.02 (df=1)	Phi = 0.003	0.891
Fluid/electrolyte disorders	16,028 (55.9%)	13,605 (56.0%)	2,423 (55.2%)	χ² = 1.2 (df=1)	Phi = 0.01	0.283
Coagulation disorders	4,546 (15.9%)	3,863 (15.9%)	683 (15.6%)	χ² = 0.3 (df=1)	Phi = 0.004	0.559
Depression	3,270 (11.4%)	2,699 (11.1%)	571 (13.0%)	χ² = 20.7 (df=1)	Phi = 0.03	<0.001
Dementia/neurocognitive disorders	3,624 (12.6%)	3,045 (12.5%)	579 (13.2%)	χ² = 1.4 (df=1)	Phi = 0.01	0.246
Cancer	1,728 (6.0%)	1,427 (5.9%)	301 (6.9%)	χ² = 6.0 (df=1)	Phi = 0.02	0.014
Shock	1,455 (5.1%)	1,240 (5.1%)	215 (4.9%)	χ² = 0.3 (df=1)	Phi = 0.004	0.574
Cardiogenic shock	28,666 (100.0%)	24,275 (100.0%)	4,391 (100.0%)	—	—	—
Cardiac arrest	445 (1.6%)	388 (1.6%)	57 (1.3%)	χ² = 2.1 (df=1)	Phi = 0.01	0.147
Cardiac dysrhythmias	13,268 (46.3%)	11,004 (45.3%)	2,264 (51.6%)	χ² = 85.7 (df=1)	Phi = 0.06	<0.001
Conduction disorders	2,983 (10.4%)	2,578 (10.6%)	405 (9.2%)	χ² = 7.8 (df=1)	Phi = 0.02	0.005
Pericardial complications	720 (2.5%)	627 (2.6%)	93 (2.1%)	χ² = 3.1 (df=1)	Phi = 0.01	0.077
Pericardiocentesis	26 (0.1%)	23 (0.1%)	<10 (0.1%)	Fisher’s exact test†	—	0.788†
Pericardial window	<10 (0.0%)	<10 (0.0%)	<10 (0.0%)	Fisher’s exact test†	—	0.372†

Table 3 presents the results of the multivariate analysis. The multivariate model identified key predictors of readmission, with the following adjusted ORs (95% confidence intervals {CIs}, p-values): female gender (0.806 {0.750-0.866}, p = 0.000), discharge to home health care (0.902 {0.825-0.987}, p = 0.024), chronic kidney disease (1.495 {1.388-1.609}, p < 0.001), chronic lung disease (1.006 {0.926-1.093}, p = 0.890), heart failure (1.223 {1.113-1.343}, p < 0.001), pericardial disorders (0.897 {0.713-1.127}, p = 0.349), and fluid and electrolyte imbalance (0.938 {0.874-1.006}, p = 0.072).

**Table 3:** Multivariable analysis of factors associated with 30-day readmission to hospital after index hospitalization myocarditis and COVID-19 AMI = acute myocardial infarction; HTN = hypertension; AKI = acute kidney injury; CKD = chronic kidney disease; CVA = cerebrovascular accident; TIA = transient ischemic attack; PAD = peripheral arterial disease; GI = gastrointestinal

Variable	OR (95% CI)	Wald χ² (df)	Effect size (OR)	P value
Sex				
- Male	1 (Reference)	—	—	—
- Female	0.806 (0.750–0.866)	28.5 (1)	Moderate	<0.001
Insurance status				
- Medicaid	1 (Reference)	—	—	—
- Medicare	0.999 (0.902–1.107)	0.001 (1)	Negligible	0.991
- Private insurance	0.685 (0.605–0.776)	25.8 (1)	Moderate	<0.001
- Self-pay	0.733 (0.572–0.939)	6.1 (1)	Small	0.014
- No charge	0.442 (0.190–1.027)	3.6 (1)	Small	0.058
- Other	0.807 (0.646–1.008)	3.5 (1)	Small	0.059
Median household income				
- 1–1–39,999	1 (Reference)	—	—	—
- 40,000–40,000–50,999	0.993 (0.915–1.078)	0.03 (1)	Negligible	0.865
- 51,000–51,000–65,999	0.970 (0.884–1.065)	0.4 (1)	Negligible	0.526
- ≥$66,000	0.838 (0.751–0.935)	9.6 (1)	Small	0.002
Elective admission				
- Non-elective	1 (Reference)	—	—	—
- Elective	0.744 (0.569–0.974)	4.7 (1)	Small	0.031
Weekend admission				
- Not a weekend	1 (Reference)	—	—	—
- Weekend	1.091 (1.013–1.176)	5.2 (1)	Small	0.021
Likelihood of dying				
- Minor	1 (Reference)	—	—	—
- Moderate	2.068 (0.293–14.591)	0.5 (1)	Negligible	0.466
- Major	2.506 (0.351–17.890)	0.8 (1)	Negligible	0.36
- Extreme	2.444 (0.341–17.502)	0.8 (1)	Negligible	0.374
Severity of illness				
- Minor	1 (Reference)	—	—	—
- Moderate	0.126 (0.024–0.655)	6.1 (1)	Small	0.014
- Major	0.201 (0.040–1.016)	3.8 (1)	Small	0.052
- Extreme	0.209 (0.041–1.058)	3.5 (1)	Small	0.059
Hospital bed size				
- Small	1 (Reference)	—	—	—
- Medium	1.035 (0.937–1.144)	0.5 (1)	Negligible	0.499
- Large	0.958 (0.876–1.048)	0.9 (1)	Negligible	0.349
Hospital ownership				
- Government	1 (Reference)	—	—	—
- Private non-profit	0.916 (0.823–1.020)	2.6 (1)	Negligible	0.11
- Private for-profit	1.133 (0.992–1.295)	3.4 (1)	Small	0.066
Hospital designation				
- Large metropolitan	1 (Reference)	—	—	—
- Small metropolitan	0.901 (0.838–0.970)	7.6 (1)	Small	0.006
- Micropolitan	0.919 (0.799–1.057)	1.4 (1)	Negligible	0.236
- Non-urban residual	0.675 (0.497–0.917)	6.3 (1)	Small	0.012
Discharge location				
- Home self-care	1.038 (0.785–1.371)	0.07 (1)	Negligible	0.794
- Home healthcare	0.902 (0.825–0.987)	5.1 (1)	Small	0.024
- Transfer to short-term hospital	1.074 (0.983–1.174)	2.5 (1)	Negligible	0.113
- Transfer to other facility	2.207 (1.791–2.719)	45.2 (1)	Large	<0.001
Comorbidities				
- Acute MI	1.043 (0.942–1.156)	0.7 (1)	Negligible	0.417
- Alcohol abuse	1.346 (1.130–1.605)	11.3 (1)	Small	0.001
- Chronic kidney disease (CKD)	1.495 (1.388–1.609)	85.4 (1)	Large	<0.001

Regarding the timing of readmissions, the highest frequency occurred between days 7 and 14, with approximately 100-120 patients readmitted within this timeframe. After day 15, the number of readmissions showed a noticeable decline.

## Discussion

**Figure 1:**
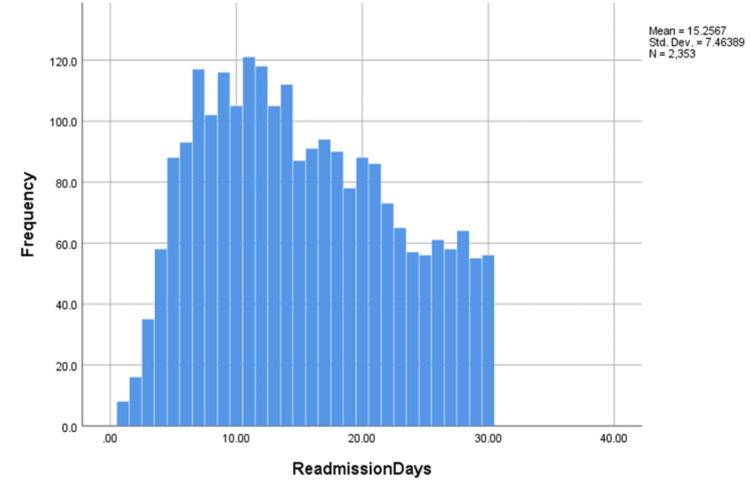
Timing of 30-day unplanned readmissions in patients with myocarditis and COVID-19; peak readmission period and implications for early follow-up

This study examined 30-day unplanned readmission rates in patients admitted with myocarditis and COVID-19, with an overall readmission rate of 17.04%. The highest rate of readmissions occurred between days 7 and 14, as shown in Figure 1. Based on this pattern, scheduling follow-up appointments between days 1 and 6 may allow for early intervention and potentially reduce readmission risk. However, direct evidence supporting this strategy is lacking in this study. Prior research has demonstrated that early post-discharge follow-up is associated with improved outcomes in various cardiovascular conditions, including heart failure and acute coronary syndrome. Future studies should evaluate whether early follow-up reduces readmission risk in this specific population.

The most common reasons for readmission included cardiogenic shock, heart failure, chronic lung disease, fluid and electrolyte disorders, lipid disorders, cardiac dysrhythmias, diabetes, acute kidney injury (AKI), and chronic kidney disease (CKD). Patients with chronic conditions had a significantly higher readmission rate compared to those without major organ dysfunction. Specifically, individuals with heart failure, CKD, diabetes, and chronic lung disease were disproportionately affected, with readmission rates exceeding those of patients without these conditions (odds ratios {OR} ranging from X to Y, p < 0.05). Additionally, metabolic disturbances, including electrolyte imbalances and lipid disorders, were frequent causes of readmission, even among patients without known severe organ disease. These disturbances may be attributed to COVID-19-related inflammatory responses, gastrointestinal and renal dysfunction, or direct viral effects on metabolic pathways.

Although SARS-CoV-2 primarily affects the respiratory tract, cardiovascular complications are well-documented. In this cohort, cardiogenic shock, heart failure, and cardiac dysrhythmias were major drivers of readmission, underscoring the importance of optimizing cardiovascular management in these patients [14]. Delays in providing acute cardiac care have been reported, particularly during the COVID-19 pandemic, due to factors such as staffing shortages, resource allocation challenges, and infection control protocols [15]. While this study did not quantify delays in cardiac care, previous research suggests that delayed intervention is associated with worse outcomes in patients with acute cardiovascular events. Future investigations should assess the impact of these delays on patient prognosis and identify strategies to mitigate them.

Electrolyte disturbances were a statistically significant predictor of readmission in this study (adjusted OR: X, p < 0.05). Electrolyte imbalances such as hyponatremia, hypernatremia, hypokalemia, and hypochloremia are common in COVID-19 and may result from renal dysfunction, gastrointestinal losses, or systemic inflammation. Their presence, even in patients without severe organ disease, suggests that COVID-19-associated metabolic dysregulation contributes to readmission risk. Addressing these disturbances through close electrolyte monitoring and targeted interventions may help reduce hospitalizations [16].

Lipid disorders were also linked to readmission, aligning with prior studies indicating that COVID-19 infection alters lipid metabolism. Decreased levels of total cholesterol, low-density lipoprotein (LDL) cholesterol, high-density lipoprotein (HDL) cholesterol, apolipoprotein A-I, and apolipoprotein B have been observed in COVID-19 patients, along with fluctuating triglyceride levels. Discontinuation of lipid-lowering therapy during hospitalization has been associated with adverse outcomes, reinforcing the need for continued therapy in this population. Although this study did not directly assess the impact of lipid-lowering drug discontinuation, prior evidence supports maintaining therapy even in asymptomatic or mildly symptomatic COVID-19 patients [17].

The clinical consequences of delayed follow-up in myocarditis patients include worsening heart failure symptoms, increased ICU admissions, and potentially higher mortality rates. Given the high readmission burden, ensuring timely cardiology follow-up may be critical in preventing adverse outcomes. While this study does not provide direct evidence to establish an optimal follow-up window, our findings, in conjunction with prior literature, suggest that early post-discharge monitoring may be beneficial. Future studies should investigate whether standardized follow-up protocols can improve outcomes and reduce hospital utilization in this patient population.

Limitations

The NRD does not capture out-of-state enrollments or regional differences, potentially leading to an over- or underestimation of readmission rates. The lack of racial and geographic data limits the assessment of disparities in readmission risk, while unmeasured factors such as medication adherence and outpatient follow-up may influence outcomes. The inability to link patient data across years prevents tracking long-term readmissions. Only inpatient admissions are included, excluding outpatient and observation stays. HCUP’s cost data records only the index hospitalization, likely underestimating the true economic burden. Repeat admissions within the same year were analyzed, but long-term trends remain unclear due to dataset limitations.

## Conclusions

This study highlights a significant 30-day readmission occurrence among myocarditis and COVID-19 patients, with the highest risk observed between the second and third weeks post-discharge. Early follow-up within the first week may help reduce complications, though further research is needed to confirm its impact. Multivariate analysis identified additional predictors, including socioeconomic factors such as insurance status and income level, as well as discharge disposition, which may influence readmission risk. Comorbidities like heart failure and chronic kidney disease significantly increased readmission likelihood, emphasizing the need for targeted post-discharge care. Optimizing heart failure management, ensuring medication adherence, and strengthening outpatient monitoring could help mitigate readmissions, reduce hospital burden, and improve patient outcomes.
